# Pyrogallol and Fluconazole Interact Synergistically *In Vitro* against Candida glabrata through an Efflux-Associated Mechanism

**DOI:** 10.1128/AAC.00100-21

**Published:** 2021-06-17

**Authors:** Dongting Yao, Guanyi Zhang, Weiqin Chen, Jia Chen, Zhen Li, Xin Zheng, Hongmei Yin, Xiaobo Hu

**Affiliations:** a Department of Laboratory Medicine, Longhua Hospital, Shanghai University of Traditional Chinese Medicine, Shanghai, China

**Keywords:** pyrogallol, fluconazole, *Candida glabrata*, efflux, resistance, synergism, pyrogallol

## Abstract

Candida glabrata is currently the first or second most commonly encountered non-*albicans Candida* species worldwide. The potential severity of *Candida* resistance mandates the discovery of novel antifungal agents, including those that can be used in combination therapies. In this study, we evaluated the *in vitro* interactions of pyrogallol (PG) and azole drugs against 22 clinical C. glabrata isolates. The potential mechanism underlying the synergism between PG and fluconazole (FLC) was investigated by the rhodamine 6G efflux method and quantitative reverse transcription (qRT)-PCR analysis. In susceptibility tests, PG showed strong synergism with FLC, itraconazole (ITC), and voriconazole (VRC), with fractional inhibitory concentration index values of 0.18 to 0.375 for PG+FLC, 0.250 to 0.750 for PG+ITC, and 0.141 to 0.750 for PG+VRC. Cells grown in the presence of PG+FLC exhibited reduced rhodamine 6G extrusion and significantly downregulated expression of the efflux-related genes *CgCDR1*, *CgCDR2*, and *CgPDR1* compared with cells grown in the presence of PG or FLC alone. PG did not potentiate FLC when tested against a Δ*Cgpdr1* strain. Restoration of a functional *CgPDR1* allele also restored the synergism. These results indicate that PG is an antifungal agent that synergistically potentiates the activity of azoles. Furthermore, PG appears to exert its effects by inhibiting efflux pumps and downregulating *CgCDR1*, *CgCDR2*, and *CgPDR1*, with *CgPDR1* probably playing a crucial role in this process.

## INTRODUCTION

Candida glabrata is among the most common non-*albicans Candida* species worldwide. Morbidity and mortality of infections caused by C. glabrata are increasing. This species can cause life-threatening nosocomial infections, especially in immunocompromised patients (1). C. glabrata exhibits intrinsically low susceptibility to azole antifungals, including fluconazole (FLC), itraconazole (ITC), and voriconazole (VRC), and frequently develops resistance on prolonged exposure to these antifungals, resulting in less effective treatment and high mortality rates (2). Thus, improvements in the antifungal activity or the development of new antifungals is urgently needed to treat C. glabrata infection. Combination treatments with antifungal and nonantifungal drugs have recently gained attention (3, 4).

In recent years, compounds extracted from natural plants (especially medicinal plants) and their chemically synthesized derivatives (such as berberine, garlic oil, and pterostilbene) have demonstrated prominent synergistic effects against *Candida* species (5–7). For instance, a natural coumarin (osthole) extracted from Fructus cnidii showed a significant synergistic effect with FLC against FLC-resistant Candida albicans by augmenting endogenous reactive oxygen species (8). Carvacrol and thymol, the principal components of thyme oil, showed a synergistic antifungal effect against C. albicans by decreasing the activities of the Cdr1 and Mdr1 efflux pumps (9). However, most studies focused on C. albicans (10), and studies on C. glabrata are rare (11).

We previously demonstrated that pyrogallol (PG; benzene-1,2,3-triol) interacted synergistically with FLC against a clinical C. glabrata isolate, but the mechanism of action remains unclear (our unpublished results). PG is a phenolic compound derived from high-molecular-weight hydrolysable tannins and can be isolated from many plant species, such as gallnuts (12). Interest has been increasing in using PG in humans and animals because of its health-promoting effects, including lung cancer prevention (13), antiatherogenic effects (important for preventing vascular diseases) (14), skin protection (15), and antiseptic and antipsoriatic activities (16). PG also has antimicrobial and antifungal activities, possibly resulting from the three hydroxyl groups in its structure (17). Its ability to boost immunity by inducing Hsp70 production makes it a potential natural protective agent (18). PG can inhibit α-glucosidase activity by binding to key active-site residues, effectively reducing the risk of cerebrovascular events (19).

Several mechanisms contribute to fungal azole resistance, among which increased expression of efflux pumps is the most significant. In C. glabrata, the major genes that induce azole resistance are *CgCDR1* and *CgCDR2*, both of which are members of the ATP-binding cassette (ABC) superfamily of efflux pump proteins (20). Data from our previous study suggested that the main basis of acquired azole resistance in C. glabrata is the constitutive upregulation of *CgCDR1* and, to a lesser extent, *CgCDR2* (21). Expression of these two transporters is regulated by the zinc finger transcription factor CgPdr1.

We hypothesized that PG lowers azole resistance in C. glabrata by influencing the functionality of efflux pumps. The objective of this study was to evaluate the *in vitro* interaction of PG in combination with different azole antifungals and to investigate the mechanism of interaction.

## RESULTS

### Synergistic activity of PG+FLC, PG+ITC, or PG+VRC against C. glabrata.

Twenty-two C. glabrata isolates were used to evaluate the anti-*Candida* activity of PG alone or together with FLC, ITC, or VRC. PG was active against all isolates, with MIC values of 16 to 64 mg/liter, and no difference was found between FLC-susceptible and FLC-resistant isolates. Combination of PG with FLC showed synergistic effects against all isolates (Table 1). The MICs in the PG+FLC group decreased by 4- to 8-fold for PG and 4- to 128-fold for FLC compared with those of each drug alone, with the fractional inhibitory concentration index (FICI) ranging from 0.188 to 0.375. PG+ITC and PG+VRC yielded similar synergistic effects. PG+ITC showed synergism against 68.2% (15/22) of isolates and no interaction against the other 31.8% (7/22). The MICs were reduced 2- to 8-fold for PG and 2- to 16-fold for ITC compared with those of each drug alone, with FICI ranging from 0.250 to 0.750. Similarly, PG+VRC showed synergism against 63.6% (14/22) of isolates and no interaction against the other 36.4% (8/22). The MICs were reduced by 4- to 8-fold for PG and 2- to 64-fold for VRC compared with those of each drug alone, with FICI ranging from 0.141 to 0.750.

**TABLE 1:** Interactions of PG with FLC, ITC, or VRC against C. glabrata clinical isolates

C. glabrata strain	MIC (μg/ml) for:				MIC (μg/ml) for combination:			FICI of^*a*^:		
	PG	FLC	ITC	VRC	PG/FLC	PG/ITC	PG/VRC	PG+FLC	PG+ITC	PG+VRC
34	32	256	8	2	8/32	8/1	4/0.25	0.375	0.375	0.250
43	32	256	8	2	8/32	8/1	4/0.25	0.375	0.375	0.250
48	32	256	4	4	8/16	4/1	4/1	0.313	0.375	0.375
49	32	256	8	4	8/16	4/2	4/0.5	0.313	0.375	0.250
52	32	256	4	2	8/2	4/0.5	4/0.03125	0.258	0.250	0.141
54	32	256	8	1	8/16	8/1	8/0.125	0.313	0.375	0.375
55	64	256	16	8	16/4	32/1	16/0.5	0.266	0.563	0.313
57	32	256	8	2	4/16	8/1	8/0.25	0.188	0.375	0.375
66	32	256	8	4	4/16	8/1	8/1	0.188	0.375	0.500
68	32	256	8	1	8/16	8/0.5	4/0.25	0.313	0.313	0.375
79	32	256	8	2	8/32	8/1	4/0.5	0.375	0.375	0.375
10	32	8	1	0.125	8/0.5	8/0.25	8/0.03125	0.313	0.500	0.500
27	32	8	0.25	0.0625	8/1	8/0.0625	4/0.03125	0.375	0.500	0.625
28	32	8	0.25	0.0625	8/1	8/0.125	4/0.03125	0.375	0.750	0.625
29	32	16	0.25	0.125	8/2	8/0.125	4/0.0625	0.375	0.750	0.625
67	32	8	0.5	0.125	8/1	4/0.25	4/0.0625	0.375	0.625	0.625
90	32	8	0.5	0.125	8/1	4/0.125	4/0.03125	0.375	0.375	0.375
115	16	8	0.5	0.0625	4/1	8/0.0625	4/0.03125	0.375	0.625	0.750
126	32	4	0.25	0.0625	4/1	16/0.0625	4/0.03125	0.375	0.750	0.625
134	32	8	0.125	0.0625	8/1	8/0.0625	8/0.03125	0.375	0.750	0.750
138	32	2	0.125	0.0625	4/0.5	8/0.03125	4/0.03125	0.375	0.500	0.625
140	32	8	0.125	0.125	8/1	8/0.03125	4/0.03125	0.375	0.500	0.375

a Synergism was defined as FICI of ≤0.5, no interaction was defined as 0.5 < FICI ≤ 4.0, and antagonism was defined as FICI of >4.0.

### PG+FLC inhibited efflux pump activity and related gene expression levels in azole-resistant C. glabrata.

**FIG 1:**
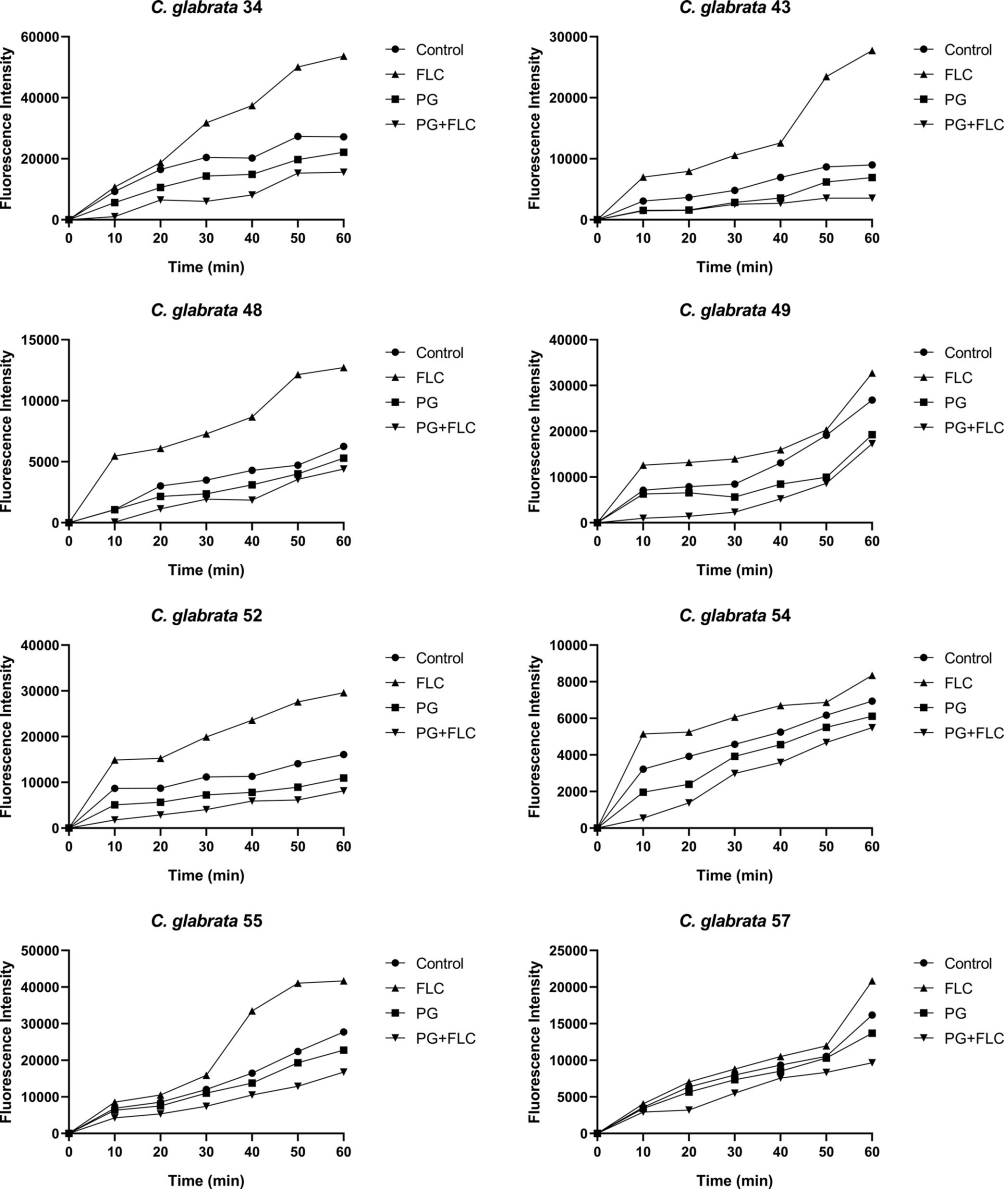
Function of the efflux pumps in 11 clinical C. glabrata isolates in the presence of PG or FLC alone or in combination at synergistic concentrations, as determined from fluorescence intensities. The fluorescence intensity reflected the amount of rhodamine 6G transported out of the cells in the presence of glucose.

The extracellular fluorescence of rhodamine 6G increased steadily over time in all isolates (Fig. 1). The fluorescence intensity of rhodamine 6G in the presence of FLC alone was higher than that in the other groups. In contrast, the fluorescence intensity of rhodamine 6G in the presence of PG alone was slightly lower than that in the control and was even lower in PG+FLC at synergistic concentrations.

**FIG 2:**
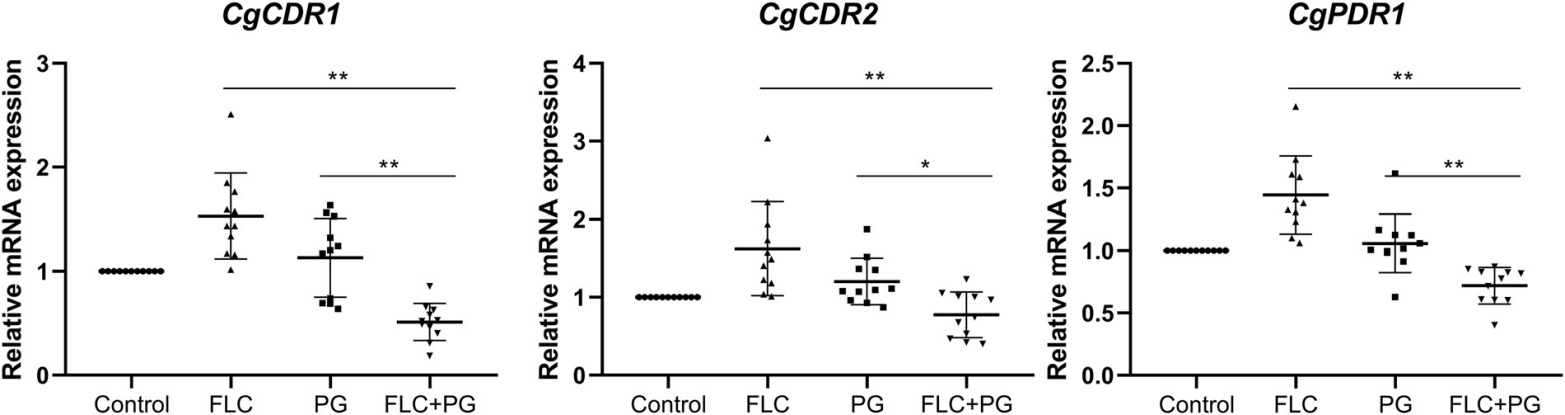
Relative *CgCDR1*, *CgCDR2*, and *CgPDR1* mRNA expression levels in 11 clinical C. glabrata isolates in the presence of PG or FLC alone or in combination at synergistic concentrations, as determined by qRT-PCR. The results shown represent the mean values of triplicate experiments. The control isolate was drug free. *, *P* < 0.05; **, *P* < 0.01.

The qRT-PCR assays showed that FLC alone significantly upregulated *CgCDR1*, *CgCDR2*, and *CgPDR1* expression compared with that in the control group in eight, nine, and nine of the isolates, respectively, whereas the corresponding expression levels in the other isolates hardly changed. The results with PG alone were inconsistent among the isolates compared with those in the control group. *CgCDR1* expression increased in four isolates, decreased in four other isolates, and was unchanged in the remaining isolates. *CgCDR2* expression increased in four isolates but remained unchanged in all other isolates. *CgPDR1* expression increased in two isolates, decreased in one isolate, and remained unchanged in all other isolates. PG+FLC significantly downregulated *CgCDR1* and *CgPDR1* expression in all isolates compared with the control group, whereas six isolates displayed *CgCDR2* downregulation, and the remaining isolates showed no significant change. In addition, compared with PG or FLC alone, PG+FLC resulted in 2.22-fold (*P* < 0.01) and 3.00-fold (*P* < 0.01) decreases in *CgCDR1* expression, respectively. Similarly, compared with PG and FLC alone, PG+FLC resulted in 1.56-fold (*P* < 0.05) and 2.10-fold (*P* < 0.01) decreases in *CgCDR2* expression and 1.47-fold (*P* < 0.01) and 2.01-fold (*P* < 0.01) decreases in *CgPDR1* expression, respectively, (Table 2, Fig. 2).

**TABLE 2:** Fold changes in *CgCDR1*, *CgCDR2*, and *CgPDR1* mRNA expression levels in clinical C. glabrata isolates, determined by qRT-PCR

C. glabrata	Fold change (mean ± SD) with:		
	FLC	PG	FLC+PG
*CgCDR1*	1.53 ± 0.41	1.13 ± 0.38	0.51 ± 0.18
*CgCDR2*	1.62 ± 0.60	1.20 ± 0.30	0.77 ± 0.29
*CgPDR1*	1.45 ± 0.31	1.06 ± 0.23	0.72 ± 0.15

### Susceptibilities and inhibitory effects on efflux pumps of PG+FLC in *CgPDR1*-disruption and -replacement mutants.

Disruption and replacement mutants were generated as described in the supplemental material. During drug-susceptibility testing, the MIC values of FLC alone were reduced in the *CgPDR1-*deficient strain C. glabrata 66/*ura3*Δ*pdr1* (Table 3). However, PG failed to enhance FLC activity against C. glabrata 66/*ura3*Δ*pdr1*, with a FICI value of 1. When *CgPDR1* was replaced, the MIC values of FLC alone recovered, and PG+FLC showed a strong synergistic effect. Similar results were obtained with PG+ITC and PG+VRC.

**TABLE 3:** Interactions of PG with azole against *CgPDR1*-deletion mutants

C. glabrata*s*train	MIC (mg/liter) for:				MIC (mg/liter) for combination:			FICI of^*a*^:		
	PG	FLC	ITC	VRC	PG/FLC	PG/ITC	PG/VRC	PA+FLC	PA+ITC	PA+VRC
66	64	256	16	8	16/32	16/1	16/0.5	0.375	0.313	0.313
66/*ura3*Δ*pdr1*	16	8	0.5	0.125	8/4	16/0.25	8/0.0625	1	1.5	1
66/*ura3*Δ*pdr1-PDR1*	64	256	16	8	16/32	16/2	8/2	0.375	0.375	0.375

a Synergism was defined as a FICI of ≤0.5, no interaction was defined as 0.5 < FICI ≤ 4.0, and antagonism was defined as a FICI of >4.0.

The fluorescence intensity of rhodamine 6G in the extracellular matrix of the *CgPDR1-*deficient strain C. glabrata 66/*ura3*Δ*pdr1* grown in the presence of FLC, alone or in combination with PG, was markedly higher than that in the other groups (Fig. 1). In contrast, the fluorescence intensity of rhodamine 6G in the extracellular matrix of the *CgPDR1*-replacement strain C. glabrata 66/*ura3*Δ*pdr1-PDR1* grown in the presence of PG+FLC at synergistic concentrations decreased to a level lower than that in the other groups.

## DISCUSSION

PG has previously been found to have an antibacterial effect against Salmonella enterica serovar Typhimurium (22), Acinetobacter baumannii (23), Pseudomonas pyocyanea, Pseudomonas putida, and Corynebacterium xerosis (24). PG showed synergistic activity with norfloxacin and gentamicin against Staphylococcus aureus (25), but the mechanism of action is unclear. In this study, our *in vitro* results indicated that, although PG alone had a limited antifungal effect (MIC, 16 to 64 mg/liter), it showed strong interaction with azole drugs, particularly FLC, against azole-resistant C. glabrata. We also tested the synergism of PG with FLC/ITC against C. albicans, Candida tropicalis, Candida parapsilosis, and Candida krusei. However, the results showed no interaction and even suggested that antagonism occurred between PG and FLC/ITC (data not shown). Thus, PG is a promising synergist in blocking cross-resistance to FLC, ITC, and VRC in C. glabrata.

C. glabrata can develop FLC resistance owing to the overexpression of ABC transporters; an approach to overcome this resistance may be to identify efflux pump inhibitors. Silva et al. (26) reported that milbemycin, an ABC transporter inhibitor, can inhibit C. glabrata efflux, shows synergy with FLC *in vivo*, and has intrinsic fungicidal activity. Transcript profiling results revealed a core of regulated genes involved in drug stress responses, including oxidoreductive processes, vesicle trafficking, and protein ubiquitination. Holmes et al. (27) found that clorgyline, a monoamine oxidase A inhibitor, acts synergistically with FLC against C. albicans and C. glabrata and inhibits rhodamine 6G efflux against an FLC-resistant C. albicans isolate. In our study, the rhodamine 6G efflux assay data clearly showed that PG inhibits the efflux of intracellular rhodamine 6G, and we infer a close association between the synergistic antifungal effects of PG+FLC and the functionality of efflux pumps in the C. glabrata isolates tested.

We evaluated the effects of PG and/or FLC on the efflux pumps and found that *CgCDR1* and *CgPDR1* were more strongly downregulated in the presence of PG+FLC in all 11 resistant C. glabrata isolates tested, whereas *CgCDR2* was slightly downregulated after PG exposure in only six resistant C. glabrata isolates. These results indicated that *CgCDR1* and *CgPDR1* played a greater role in the resistance than *CgCDR2*. We also found that efflux of intracellular rhodamine 6G and the mRNA expression levels of *CgCDR1*, *CgCDR2*, and *CgPDR1* were higher in most isolates in the presence of FLC alone than in the control group. FLC, a known substrate of the efflux pump, may stimulate the expression of efflux pump genes, leading to enhanced efflux. When characterizing Δ*pdr1* derivatives of C. glabrata, we found that the synergistic effects of PG with azoles disappeared when *CgPDR1* was disrupted and that these effects recovered when *CgPDR1* was replaced. These findings indicate that PG exerted a synergistic effect through *CgPDR1*. Furthermore, PG showed no synergism with FLC or ITC against other *Candida* species, which may imply the potential role of *CgPDR1*.

Despite these promising results, at high doses, PG may cause cytotoxicity because of an imbalance between oxidants and antioxidants, limiting its application. The 50% lethal dose of PG is 1,600 mg/kg in rabbits (28) and 862 mg/kg in mice (29). In a 3-month study, mice and rats were administered PG at doses of up to 600 and 150 mg/kg, respectively, 5 days per week for up to 14 weeks (30). All mice survived, most rats survived, and their body weights were comparable with those of the controls. In a 2-year dermal study, no evidence of carcinogenic activity was found in F344/N rats administered 5, 20, or 75 mg/kg PG 5 days per week for up to 104 weeks (31). Defoirdt et al. (32) reported that pyrogallol protects giant river prawn larvae and brine shrimp from pathogenic Vibrio harveyi, while showing relatively low toxicity. Even then, identifying appropriate strategies to reduce the toxicity of PG, such as limiting the dose and looking for side-effect-counteracting agents, is essential. Natural antioxidants, such as resveratrol and silymarin (33, 34), have been reported to attenuate PG-induced toxicity and are primarily used as dietary supplements because of their relative nontoxicity, where even minor dosage errors are not expected to produce negative effects (35). Recent developments in pharmacology and toxicology have made the evaluation of PG efficacy and toxicity more reliable and convenient, which may lead to an expansion of PG in clinical applications.

In conclusion, our observations suggest that PG participates in lowering efflux pump activity by downregulating the expression of *CgCDR1*, *CgCDR2*, and *CgPDR1* to produce a *CgPDR1-*dependent effect.

In future experiments, more FLC-resistant clinical isolates will be analyzed, and DNA sequencing will be performed to decipher the associated molecular mechanisms. Further *in vivo* studies are needed to support clinical applications.

## MATERIALS AND METHODS

### Strains.

Twenty-two clinical C. glabrata isolates (11 FLC-resistant and 11 FLC-susceptible isolates) and C. glabrata *66 CgPDR1*-disruption and -replacement mutants were used. All strains were routinely stored at −80°C in yeast-peptone-dextrose liquid medium (1% yeast extract, 2% peptone, and 2% dextrose), supplemented with 30% (vol/vol) glycerol, and recultured at least twice on Sabouraud agar (Kehua Biotech Co., Shanghai, China) at 35°C before use in the experiments.

### Chemicals.

FLC (National Institutes for Food and Drug Control [NIFDC], Beijing, China), ITC (NIFDC), VRC (Haisi Co., Jincheng, Shanxi, China), and PG (U-sea Biotech, Shanghai, China) were obtained commercially. The purity of PG (>99.90%) was confirmed via high-performance liquid chromatography. FLC was prepared in sterile distilled water at 5,000 mg/liter. ITC was dissolved in dimethyl sulfoxide (DMSO) at 5,000 mg/liter. VRC was prepared in a dedicated solvent (ethanol and propylene glycol, 1:1) at 2,000 mg/liter. PG was prepared in DMSO at 10,000 mg/liter. All stock solutions were stored at −20°C.

### Antifungal activities of PG alone and in combination with FLC, VRC, and ITC.

The MICs of PG+FLC, PG+VRC, and PG+ITC against C. glabrata strains were tested using broth microdilution checkerboard assays based on Clinical and Laboratory Standards Institute standard M27-A3 (36). The MICs alone and in combination were defined as 50% of inhibition compared with the growth control. MICs were read visually. The drugs tested were serially diluted 2-fold in RPMI 1640 medium (Invitrogen, Carlsbad, CA, USA) as previously described (37). The final concentrations were 4 to 256 mg/liter for PG, 2 mg/liter to 1.024 g/liter for FLC, 0.125 to 64 mg/liter for ITC, and 0.125 to 64 mg/liter for VRC. A 50-μl aliquot of each PG dilution and 50 μl of RPMI 1640 medium were added to individual wells in 96-well plates (Corning, Inc., Corning, NY, USA) in the first columns, and a 50-μl aliquot of each azole drug dilution and 50 μl of RPMI 1640 medium were added to row H. The well at the intersection of column 1 and row H was drug free and served as a control. Then, 50-μl aliquots of a PG-dilution series or an azole drug-dilution series were added to columns 2 to 11 and lines A to G, respectively. Next, 100 μl of cells was added to each well at a final concentration of 0.5 to 2.5 × 10^3^ cells/ml, except for column 12, to which 200 μl of RPMI 1640 medium was added as a negative control. The plates were incubated at 35°C for 24 or 48 h. Drug interactions were analyzed based on the FICI, calculated as MIC(A) combined/MIC(A) alone plus MIC(B) combined/MIC(B) alone. Synergism was defined as a FICI of ≤0.5, no interaction was defined as 0.5 < FICI ≤ 4.0, and antagonism was defined as a FICI of >4.0 (38). The experiments were performed in duplicate.

### Rhodamine 6G efflux assay.

The rhodamine 6G efflux assay was performed as previously described (21), with a few modifications. Isolates were incubated at 37°C overnight without any drug or with PG alone, FLC alone, or PG+FLC at synergistic concentrations. Isolates were cultured overnight, then adjusted to a cell density of 5 × 10^7^ cells/ml in phosphate-buffered saline (PBS) and incubated at 37°C for 4 h in an orbital shaker (180 rpm; Yiheng Biotech, Shanghai, China). Rhodamine 6G was added at a final concentration of 10 mM, and the cultures were incubated at 37°C for 2 h. After the cells were washed twice with sterile PBS, glucose was added at a final concentration of 4 mM, and the cultures were shaken at 30°C for 1 h. During this period, the suspension was centrifuged at 3,000 × *g* every 10 min, and 100 μl of the supernatant from each group was transferred to individual wells of 96-well plates. The rhodamine 6G fluorescence in each sample was measured using a BioTek Synergy H4 microplate reader (BioTek Instruments, Winooski, VT, USA). The excitation and emission wavelengths were 515 and 555 nm, respectively.

### Gene expression analysis.

The qRT-PCR analysis was performed as described previously (21), with minor modifications. Isolates were incubated without any drug or with PG alone, FLC alone, or PG+FLC at synergistic concentrations at 37°C overnight. The suspensions were adjusted to 5 × 10^7^ cells/ml in PBS, and the supernatants were collected after centrifugation at 3,000 × *g*. Total RNA was isolated using a yeast RNAiso reagent kit (TaKaRa, Shiga, Japan) according to the manufacturer’s instructions. RT-PCR was performed using RevertAid first-strand cDNA synthesis kits (Thermo Fisher Scientific, Waltham, MA, USA). qRT-PCRs for *CgCDR1*, *CgCDR2*, and *CgPDR1* were run in triplicate using SYBR green real-time PCR master mix kits (Toyobo, Osaka, Japan) in an ABI 7500 real-time fluorescent quantitative PCR system (Applied Biosystems, Foster City, CA, USA). The primers used in this study are listed in Table 4. Each qRT-PCR mixture (25 μl) contained 12.5 μl SYBR green real-time PCR master mix, 9.5 μl double-distilled water, 2 μl each primer, and 1 μl cDNA. PCR conditions were as follows: initial denaturation at 95°C for 1 min, followed by 40 cycles of 15 s at 95°C, 15 s at 60°C, and 45 s at 72°C. Target gene expression was quantified using the 2^–ΔΔCT^ method, with *ACT1* as a control (39).

**TABLE 4:** Primers used for qRT-PCR in this study

Primer	Sequence
CgCDR1F	5′-ACACCAACAACAGCATCT-3′
CgCDR1R	5′-ATTCTCCGCTTACCTACG-3′
CgCDR2F	5′-CAACGCTATGAGGGAAAA-3′
CgCDR2R	5′-AACATAAGTGGCGTGGGT-3′
CgPDR1F	5′-AGCCTTGCCGATAGTCATAC-3′
CgPDR1R	5′-AGGTCAGGGCATACTTCAG-3′
ACT1F	5′-AGAAGTTGCTGCTTTAGTT-3′
ACT1R	5′-GACAGCTTGAATGGAAAC-3′

### Statistical analysis.

Results are reported as the mean ± standard deviation (*n *= 3) and were calculated using IBM SPSS Statistics, version 24.0 (IBM Corp., Armonk, NY, USA). Differences among groups were analyzed using one-way analysis of variance, with the least-significant difference method. A *P* value of <0.05 was considered to reflect a statistically significant difference.

### Data availability.

GenBank accession numbers of the molecular identification of the strains are MW709447 to MW709456 (https://www.ncbi.nlm.nih.gov/nuccore/?term=MW709447:MW709456[accn]) and MW729709 to MW729720 (https://www.ncbi.nlm.nih.gov/nuccore/?term=MW729709:MW729720[accn]).
