# Supplementary material for: Pyrogallol and Fluconazole Interact Synergistically In Vitro against Candida glabrata through an Efflux-Associated Mechanism
Source: Antimicrob Agents Chemother. 2021 Jun 17;65(7):e00100-21. doi: 10.1128/AAC.00100-21 (PMC8373228; doi:10.1128/AAC.00100-21)
Supplement: Supplemental file 1 — Supplemental material. Download AAC.00100-21-s0001.pdf, PDF file, 0.2 MB [file aac.00100-21-s0001.pdf]

Supplemental Material

***CgPDR1* disruption and replacement**

The promoter-dependent disruption of genes (PRODIGE) method for PCR product-mediated gene disruption was employed (1, 2). Briefly, *ura3* mutants derived from *C. glabrata* 66 were isolated via selection on 5-fluoroorotic acid. We designated this mutant as *C. glabrata* 66/*ura3*. PCR products containing the *URA3* coding sequence from the plasmid template pRS416 plus each of the 60-bp upstream and downstream sequences of *CgPDR1* generated with long primers (PDR1-URA3F−PDR1-URA3R) were used to transform the *C. glabrata* 66/*ura3* strain. Following selection on synthetic dextrose minimal medium, transformants were screened using PCR. Loss of the PDR1uF-PDR1iR product and generation of the PDR1uF-URA3iR product confirmed *CgPDR1* disruption (Table 1; Fig. 1). The *CgPDR1*-deficient mutant was designated as *C. glabrata* 66/*ura3pdr1Δ*. For *CgPDR1* replacement, a PCR product representing the *CgPDR1* coding sequence plus 430–680 bp upstream and downstream sequence was amplified from *C. glabrata* 66 genomic DNA (primers PDR1uF-PDR1dR). These products were used to transform the *C. glabrata* 66/*ura3pdr1Δ* strain, and the transformed colonies were selected on yeast peptone dextrose plates containing 1 mg/L cycloheximide. Colonies were screened as described above with the primer pair PDR1uF2-PDR1iR (Table 1; Fig. 1). The mutant with replaced *CgPDR1* was designated as *C. glabrata* 66/*ura3pdr1Δ-PDR1*.

Table S1 Primers used to generate mutants

PRODIGE-based gene disruption	
PDR1-URA3F	5′-GCCTTTTTTTTTAGAATATATTGGTAAAGTCATTCTTTAGC TACGTTATTGAGAGAATATGTCGAAAGCTACATATAAGG-3′
PDR1-URA3R	5′-TGATTTTTCAGATTAAATATAAAATTATACAGGCTATGCA CACTGTCTAAATTAATAGCATTAGTTTTGCTGGCCGCATC-3′
PCR screening and PDR1 replacement	
PDR1uF	5′-GGCGTATTCATAGAATCCGAA-3′
PDR1iR	5′-CCATAGTATTCGTCGAGAGCA-3′
URA3iR	5′-CAGCAACAGGACTAGGATGAG-3′
PDR1dR	5′-GACCTCTGTGAAAAGCTACTG-3′
PDR1uF2	5′-GGTCCTTCTAATAGTCATCTTT-3′

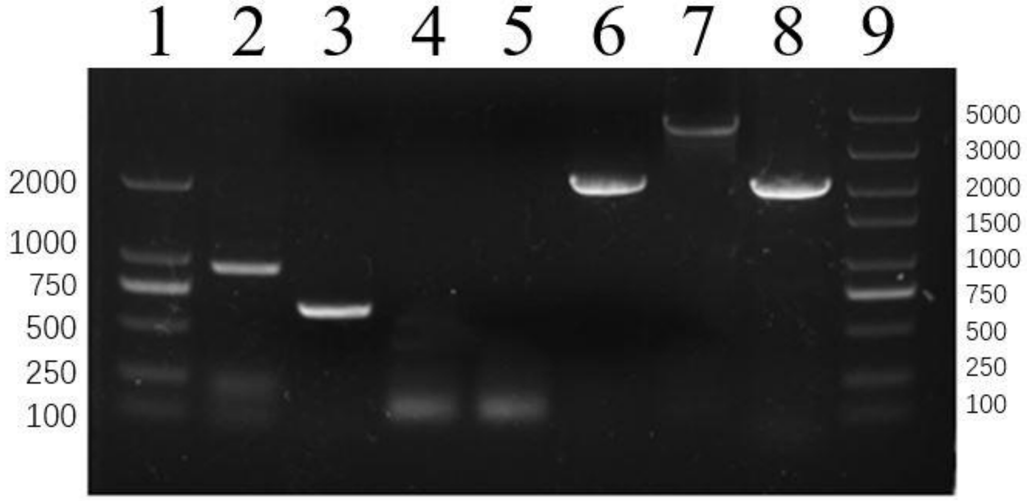

Fig S1 PCR screen of *CgPDR1* disruption and *CgPDR1* replacement. Lanes 1, 9: size marker. Lane 2: PCR products of PDR1-URA3F–PDR1-URA3R (922 bp) from plasmid template pRS416 prepared for *PDR1* disruption. Lanes 3, 4: *C. glabrata* 66/*ura3 pdr1Δ*; formation of the PDR1uF-URA3iR product (lane 3, 623 bp) and loss of the PDR1uF-

PDR1iR product (lane 4) confirmed *CgPDR1* disruption. Lanes 5, 6: *C. glabrata* 66; loss of the PDR1uF-URA3iR product (lane 5) and formation of the PDR1uF-PDR1iR product (lane 6, 2,097 bp) as control. Lane 7: PCR products of PDR1uF-PDR1dR (4,448 bp) from *C. glabrata* 66 prepared for *CgPDR1* replacement. Lane 8: *C. glabrata* 66/*ura3pdr1Δ-PDR1*; formation of the PDR1uF2-PDR1iR product (2,142 bp) confirmed replacement of *CgPDR1*.

References

1. Edlind TD, Henry KW, Vermitsky J-P *et al.* 2005. Promoter-dependent disruption of genes: simple, rapid, and specific PCR-based method with application to three different yeast. Curr Genet 48:117–125.
2. Vermitsky J, Earhart KD, Smith WL *et al.* 2006. Pdr1 regulates multidrug resistance in *Candida glabrata*: gene disruption and genome-wide expression studies. Mol Microbiol 61:704–722.
